# A New Method of Preserving Raw Meat

**Published:** 1881-02

**Authors:** 


A NEW METHOD OF PRESERVING RAW MEAT.—A new and apparently most valuable method of preserving raw meat, discovered by Prof. Artimini, of Florence, bids fair to supply a long-felt want, and to have an appreciable effect upon our markets According to a report by Professors Barff and Mills, of Glasgow, and Dr. Stevenson, of Guy's Hospital, meat six months old was found to be perfectly sound and good, the muscular fibres unchanged, and the nutritive properties unimpaired. The material employed is stated to be less expensive than salt, and not only wholesome but pleasant to the taste.—*Medical Times and Gazette.*

